# Preoperative Assessment and Management of Cardiovascular Risk in Patients Undergoing Non-Cardiac Surgery: Implementing a Systematic Stepwise Approach during the COVID-19 Pandemic Era

**DOI:** 10.3390/jcdd8100126

**Published:** 2021-10-03

**Authors:** Eduardo Bossone, Filippo Cademartiri, Hani AlSergani, Salvatore Chianese, Rahul Mehta, Valentina Capone, Carlo Ruotolo, Imran Hayat Tarrar, Antonio Frangiosa, Olga Vriz, Vincenzo Maffei, Roberto Annunziata, Domenico Galzerano, Brigida Ranieri, Chiara Sepe, Andrea Salzano, Rosangela Cocchia, Massimo Majolo, Giuseppe Russo, Giuseppe Longo, Mario Muto, Paolo Fedelini, Ciro Esposito, Alessandro Perrella, Gianluca Guggino, Eliana Raiola, Mara Catalano, Maurizio De Palma, Luigia Romano, Gaetano Romano, Ciro Coppola, Ciro Mauro, Rajendra H. Mehta

**Affiliations:** 1 Cardiology Division, A. Cardarelli Hospital, 80131 Naples, Italy; sasichian@gmail.com (S.C.); caponevalentina92@libero.it (V.C.); annunziata.robert@gmail.com (R.A.); makida@alice.it (C.S.); rosangela.cocchia@aocardarelli.it (R.C.); ciro.mauro@aocardarelli.it (C.M.); 2 Cardiovascular Imaging Center, IRCCS SDN, 80143 Naples, Italy; cademartirifilippo@gmail.com (F.C.); ranieribrigida@gmail.com (B.R.); andre.salzano@gmail.com (A.S.); 3 Cardiac Centre, King Faisal Specialist Hospital and Research Center, Riyadh 11211, Saudi Arabia; hani@kfshrc.edu.sa (H.A.); olgavriz@yahoo.com (O.V.); domenicogalzerano@libero.it (D.G.); 4 ProMedica Monroe Regional Hospital, Monroe, MI 48162, USA; rahul34mehta@gmail.com; 5 Vascular Surgery Unit, A. Cardarelli Hospital, 80131 Naples, Italy; carlo.ruotolo@aocardarelli.it; 6 Henry Ford Allegiance Hospital, 205 North East Avenue, Jackson, MI 49201, USA; tarar_88@hotmail.com; 7 Post Operative Intensive Care Division, A. Cardarelli Hospital, 80131 Naples, Italy; antonio.frangiosa@aocardarelli.it (A.F.); vincenzo.maffei@aocardarelli.it (V.M.); 8 Health Management Office, A. Cardarelli Hospital, 80131 Naples, Italy; massimo.majolo@aocardarelli.it (M.M.); direzione.sanitaria@aocardarelli.it (G.R.); eliana.raiola@aocardarelli.it (E.R.); ciro.coppola@aocardarelli.it (C.C.); 9 Healthcare Direction, A. Cardarelli Hospital, 80131 Naples, Italy; direzione.generale@aocardarelli.it; 10 Diagnostic and Interventional Neuroradiology, AORN Antonio Cardarelli, 80131 Naples, Italy; mario.muto@aocardarelli.it; 11 Urology Department, Antonio Cardarelli Hospital, 80131 Naples, Italy; paolo.fedelini@aocardarelli.it; 12 Liver Intensive Care Unit, AORN A. Cardarelli, 80131 Naples, Italy; ciro.esposito@aocardarelli.it; 13 Infectious Disease of Healthcare Direction, AORN Antonio Cardarelli, 80131 Naples, Italy; alessandro.perrella@aocardarelli.it; 14 Thoracic Surgery Unit, Antonio Cardarelli Hospital, 80131 Naples, Italy; gianluca.guggino@aocardarelli.it; 15 Nuclear Medicine Department “Cardarelli” Hospital Naples, 80131 Naples, Italy; mara.catalano@aocardarelli.it; 16 UOC Chirurgia Generale 2, AORN Cardarelli, 80131 Naples, Italy; maurizio.depalma@aocardarelli.it; 17 Department of General and Emergency Radiology, “Antonio Cardarelli” Hospital, Antonio Cardarelli St. 9, 80131 Naples, Italy; luigia.romano@aocardarelli.it; 18 Orthopedic Unit, Antonio Cardarelli Hospital, 80131 Naples, Italy; gaetano.romano@aocardarelli.it; 19 Duke Clinical Research Institute, Durham, North Carolina, 300 W Morgan St, Durham, NC 27701, USA; mehta007ster@gmail.com

**Keywords:** non-cardiac surgery, perioperative cardiovascular management, COVID-19, teleconsulting

## Abstract

Major adverse cardiac events, defined as death or myocardial infarction, are common causes of perioperative mortality and major morbidity in patients undergoing non-cardiac surgery. Reduction of perioperative cardiovascular risk in relation to non-cardiac surgery requires a stepwise patient evaluation that integrates clinical risk factors, functional status and the estimated stress of the planned surgical procedure. Major guidelines on preoperative cardiovascular risk assessment recommend to establish, firstly, the risk of surgery per se (low, moderate, high) and the related timing (elective vs. urgent/emergent), evaluate the presence of unstable cardiac conditions or a recent coronary revascularization (percutaneous coronary intervention or coronary artery bypass grafting), assess the functional capacity of the patient (usually expressed in metabolic equivalents), determine the value of non-invasive and/or invasive cardiovascular testing and then combine these data in estimating perioperative risk for major cardiac adverse events using validated scores (Revised Cardiac Risk Index (RCRI) or National Surgical Quality Improvement Program (NSQIP)). This stepwise approach has the potential to guide clinicians in determining which patients could benefit from cardiovascular therapy and/or coronary artery revascularization before non-cardiac surgery towards decreasing the incidence of perioperative morbidity and mortality. Finally, it should be highlighted that there is a need to implement specific strategies in the 2019 Coronavirus disease (COVID-19) pandemic to minimize the risk of transmission of COVID-19 infection during the preoperative risk assessment process.

## 1. Introduction

Cardiovascular risk of patients undergoing non-cardiac surgery results from an interplay of patient (i.e., age, comorbidities, functional capacity) and surgery-specific factors (i.e., type, timing and duration of surgery, duration of anesthesia) [1,2,3,4,5]. In European countries, 167,000 cardiovascular events occur annually in patients undergoing non-cardiac surgical procedures, of which 19,000 are life-threatening [1,2,3,4,5]. Furthermore, the ongoing 2019 Coronavirus disease (COVID-19) pandemic has created new and unpredictable challenges for modern medicine and healthcare systems, also concerning the appropriate methodology to prevent transmission of severe acute respiratory syndrome coronavirus-2 (SARS-CoV-2) during cardiac consultation and perioperative management [6,7,8,9].

**Figure 1:**
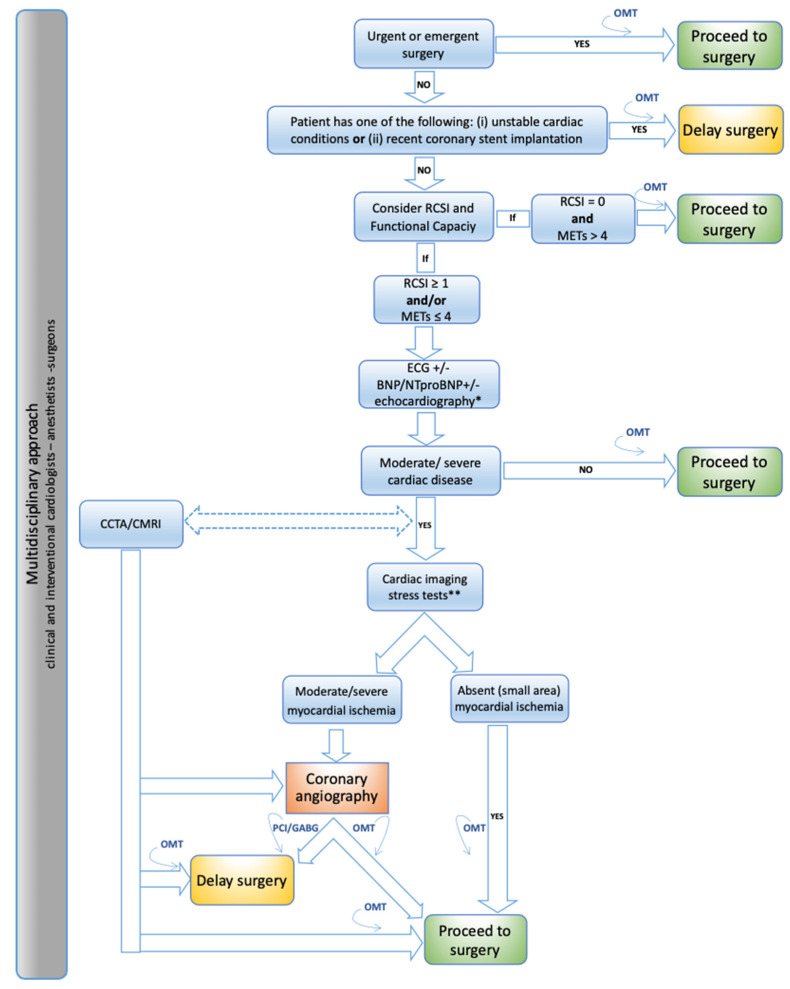
Systematic stepwise approach to the preoperative assessment and management of cardiovascular risk in patients undergoing non-cardiac surgery [1,2]. BNP, brain natriuretic peptides; CCTA, coronary computed tomography angiography; CMR, cardiac magnetic resonance; CV, cardiovascular; ECG, electrocardiogram; METs, metabolic equivalents; NT-proBNP, N-terminal prohormone of brain natriuretic peptides; OMT, optimal medical therapy; RCSI, revised cardiac index score; TTE, transthoracic echocardiography*. Handheld echocardiography +/− comprehensive TTE ** SPECT, stress echocardiography, stress CMR.

The aim of this review is to present a systematic stepwise approach to the preoperative assessment and management of cardiovascular risk in patients undergoing non-cardiac surgery and to discuss the related implementation during the COVID-19 pandemic based on current scientific evidence and guidelines (Figure 1, Table 1).

## 2. Step 1: Consider the Timing of Surgery

According to intervention timing, surgery can be classified into three main categories: emergent (to be performed within 6 h), urgent (to be performed within 6–24 h) and elective (to be performed within days or even planned) [1,2,3,4,5]. In urgent/emergent surgeries, the role of the cardiology consultant is to provide recommendations on perioperative medical management, minimization of the cardiovascular risks and surveillance for cardiovascular events, usually without performing additional tests to avoid any delay in the surgery [1,2,3,4,5]. Conversely, in elective procedures, a comprehensive clinical assessment of the patient is required (including non-invasive and/or invasive testing if needed) in order to define the overall surgical cardiovascular risk and implement appropriate therapeutic interventions [1,2,3,4,5].

## 3. Step 2: Identify Unstable Cardiac Conditions and Recent Coronary Revascularization

### 3.1. Unstable Cardiac Conditions

Patients undergoing elective surgery should be carefully assessed in order to exclude the presence of unstable cardiac conditions, namely acute coronary syndrome, acute decompensated heart failure (HF), tachyarrhythmias or bradyarrhythmias associated with hypotension or requiring urgent medical attention (i.e., ventricular tachycardia or high-grade atrioventricular block) and symptomatic severe valvular disease [1,2]. In this case, the surgery should be delayed in order to evaluate and to treat the patient as appropriate [1,2].

### 3.2. Recent Coronary Stent Implantation

A recent coronary stent implantation needs to be ruled out considering that a premature cessation of dual antiplatelet therapy (DAPT) is associated with a high risk for ischemic events [10,11,12]. In fact, a minimum period of DAPT after percutaneous coronary intervention (PCI) is required in order to allow vascular healing and progressive stent endothelialization and, consequently, to prevent stent-related thrombotic complications [10,11,12,13]. Based on this assumption, Rossini et al. suggest that elective surgery requiring discontinuation of the P2Y12 inhibitor be delayed at least 1 month after bare metal stent (BMS) implantation and 3 months after drug eluting stent (DES) implantation [13]. In the case of not deferrable surgery, a multidisciplinary consultation should be implemented in order to appropriately guide the clinical decision-making process [2,13]. As opposed to this, asymptomatic patients who underwent CABG within the previous six years are relatively protected from myocardial infarction complicating non-cardiac surgery and may undergo non-cardiac surgery without routine preoperative stress testing [1].

## 4. Step 3: Assess the Functional Capacity and the Cardiovascular Perioperative Risk

After excluding unstable cardiac conditions and recent coronary stent implantation, patient’s functional capacity and cardiovascular perioperative risk need to be assessed. In this regard, specific available tools may be implemented such as the Revised Cardiac Risk Index (RCRI) and the National Surgical Quality Improvement Program (NSQIP) universal surgical risk calculator [1,14,15].

### 4.1. Functional Capacity

Functional capacity, commonly measured in metabolic equivalents (METs), represents a pivotal step of the preoperative cardiac risk assessment algorithm [1,2,3,4]. It should be underscored that, among patients with overt cardiorespiratory disease as well as apparently healthy subjects, functional capacity has been demonstrated to be a stronger predictor of an increased risk of death as compared with associated clinical features or risk factors (hypertension, smoking and diabetes) or other exercise-test indices (ST-segment depression, peak heart rate and the development of arrhythmias during exercise) [16,17,18] (Table 2).

### 4.2. Cardiovascular Perioperative Risk Scores 

Perioperative major adverse cardiac events (MACEs) may be accurately predicted by cardiovascular risk scores, namely the RCRI and the NSQIP [14,15]. The RCRI, the simplest and most clinically used score, includes six variables (1 point is assigned to each variable): history of ischemic heart disease, history of cerebrovascular disease, history of congestive HF, preoperative treatment with insulin, preoperative serum creatinine level ≥ 2 mg/dL and high-risk procedure [14]. 

A detailed classification of surgical interventions according to surgical risk (low, intermediate or high) is reported in Supplementary Table S1. 

The NSQIP may provide superior predictive discrimination, but it results to be more complicated to be implemented [15].

### 4.3. Combining Functional Capacity and Cardiovascular Perioperative Risk Score

Combining functional capacity and cardiovascular perioperative risk score represents a decision-making crossroad of the perioperative management pathway [1,2,3,4]. In asymptomatic patients with moderate or good functional capacity (RCRI = 0 and ≥4 METs), additional tests are unlikely to change perioperative management. Thus, it is appropriate to directly refer the patient for surgery. On the other hand, in patients with low (≤4 METs) or not assessable functional capacity and/or RCRI ≥1, additional tests are advisable (Figure 1).

## 5. Step 4: Measuring Blood Biomarkers Levels and Performing Electrocardiogram/Cardiac Imaging Tests

Serum biomarkers, electrocardiogram (ECG) and non-invasive imaging cardiac tests may represent an important added diagnostic/prognostic value in the evaluation of patients with RCRI ≥ 1 and/or <4 METs [1,2,3]. It should be underscored that to decrease the risk of patient to patient, patient to imager and imager to patient SARS-CoV-2 contamination, the indication for any cardiac imaging test should be carefully evaluated [6,7,9] (Table 1).

### 5.1. Biomarkers: BNP/NT-ProBNP and Troponin

The 2016 ‘Canadian Cardiovascular Society Guidelines on Perioperative Cardiac Risk Assessment and Management for Patients Who Undergo Noncardiac Surgery’ recommend measuring brain natriuretic peptide (BNP) or the N-terminal (NT)-pro hormone BNP (NT-proBNP) (to be preferred in patients taking sacubitril-valsartan) levels before surgery to enhance perioperative cardiac risk stratification in patients with elevated risk (i.e., patients who are 65 years of age or older as well as in patients who are 45–64 years of age with a RCRI score ≥ 1) [3,19].

In addition, daily troponin measurements along with ECG are currently indicated for 48–72 h after surgery among patients with overt/suspected coronary artery disease (CAD) and/or abnormal preoperative natriuretic peptide values (BNP >9 2 ng/L or NT-pro BNP > 300 ng/L) [3,20].

### 5.2. ECG

Routine preoperative resting ECG needs to be performed among patients with an RCRI score ≥ 1 or low functional capacity (<4 METs) [1,2]. The cardiology consultant may also consider performing ECG in patients with overt cardiovascular risk factors and/or diseases undergoing low-risk surgery presenting [1,2].

In this regard, ECG signs of potential underlying heart disease [i.e., arrhythmias such as atrial fibrillation (AF)/flutter and premature ventricular complexes, left ventricular hypertrophy, left bundle branch block and ischemic alterations] should be carefully searched and interpreted in the light of patients’ clinical features [1,2].

### 5.3. Resting Transthoracic Color-Doppler Echocardiography

Resting transthoracic color-Doppler echocardiography (TTE) is the most readily available and versatile tool for evaluating heart structure and function [21]. It should be underlined that routine TTE is not indicated during the preoperative cardiovascular assessment [1,2]. However, it may be useful in patients with intermediate/high surgical risk (RCRS ≥ 1) and/or with poor functional capacity (<4 METs) [1,2]. Due to the COVID-19 pandemic it is safe to undertake a bedside quick focused cardiac ultrasound study (FoCUS) according to standardized but restricted clinically driven protocol in order to reduce patient contact with the machine as well as with the clinician performing the test [6,7,9]. In this regard, handheld ultrasound devices are being increasingly used in clinical practice today [6,9,22] (Table 1).

### 5.4. Non-Invasive Imaging Stress Testing for Ischemic Heart Disease

Non-invasive cardiac imaging stress testing (stress echocardiography, gated single-photon emission computed tomography (SPECT) and stress cardiac magnetic resonance imaging (CMRI)) is usually implemented among patients with poor functional capacity (<4 METs) candidate to intermediate-high risk non-cardiac surgery (RCRI ≥ 1) in order to detect the presence and to define the extent of stress-inducible ischaemia [1,2,3,4]. A negative test is associated with low incidence of perioperative cardiac events (high negative but low positive predictive value (between 25 and 45%)) [1,2,3,4]. The type of stress testing used should be chosen by the patient’s clinical characteristics along with local availability and expertise. Virtually all patients who are anticipated to reach a satisfactory workload should undergo an exercise stress test (treadmill, recumbent bicycle) rather than a pharmacologic stress test (vasodilators such as dipyridamole and adenosine or inotropes as dobutamine) [1,2,5]. It has to be emphasized that the performance of exercise testing has major limitations in the SARS-CoV-2 pandemic as during exercise there is “de facto” a higher exposition to COVID-19 as a direct consequence of the increased patient breath rate along with the amount of aerosol or droplet production [6,7]. In this regard, pharmacologic rather than physical stress should be preferred. Coronary computed tomographic angiography (CCTA) remains an additional option to be considered [6,7].

### 5.5. Coronary Angiography 

Indications for preoperative conventional coronary angiography (CA) are confined to a highly selected patient cohort and reflect those in the non-surgical setting [1,2,3,4,11,12,23,24]. Specifically, the 2014 ‘’ESC/ESA Guidelines on non-cardiac surgery: cardiovascular assessment and management’ recommend CA in unstable patients scheduled for elective surgery (class of recommendation I A) [1]. CA should also be performed in unstable patients scheduled for urgent surgery (class of recommendation IIa C). Furthermore, it has to be underscored that in selected stable patients (RCRI ≥ 1 and/or ≤4 METS) CA is usually preceded and triggered by an imaging stress test positive for moderate/severe ischemia [1,2] (Figure 1). However, the decision to proceed to CA is up to a multidisciplinary consensus among cardiologists, anesthesiologists and surgeons, taking into account the single patient based comprehensive clinical scenario.

### 5.6. Coronary Computed Tomographic Angiography

CCTA prior to non-cardiac surgery remains uncertain. [1] However, CCTA reducing the potential COVID-19 exposure time between health care personnel and patients may be preferred over CA or even other non-invasive imaging modalities to assess CAD severity [7].

However, in every day clinical practice, most patients undergoing major non-cardiac surgery have to perform a contrast enhanced CT exam for vascular assessment (i.e., CT angiography) and/or for oncologic purposes (i.e., staging, restaging and so forth). With currently available state-of-the-art CT technology it really does not make a big difference to include a proper CCTA within a contrast enhanced CT performed for other reasons. It does not require a significant increase in radiation exposure or contrast material administration. Of course, this is a very demanding proposition under technical and logistical aspects. We would call this an “opportunistic” application of CCTA for risk stratification (Figure 1). This is not just an additional exam, but it is the actual improvement of cardiovascular risk stratification in patients undergoing surgery and high-risk procedures. There should be a careful evaluation of patients who would not adequately benefit from this assessment. On the other hand, the technological development of current and next generation CT equipment will progressively make it more and more easy to obtain this kind of information. At some point in time, the adequate visualization of heart and coronary arteries will become just “collateral” information already included in the CT package. Of course, this will completely change the way we look at our patients. In addition, the potential to guide the pharmacological management of these patients during and after the procedure is quite relevant [25].

### 5.7. Cardiac Magnetic Resonance Imaging

Stress CMRI can be employed for risk stratification to assess myocardial ischemia through the injection of the gadolinium contrast agent at rest and after administration of a pharmacological stressing agent, either positive inotropic/chronotropic or vasodilator agents [26] (Figure 1). Reduced blood supply at rest is seen in patients with myocardial ischemia or fibrosis while impaired first pass of the contrast agent performed during pharmacological stress could detect flow-limiting coronary artery stenosis [27]. In addition, amongst cardiac masses, low blood supply could help to discriminate mural thrombi from other masses [26]. Lastly, due to its accuracy and reproducibility, CMRI could provide reliable information on cardiac morphology and masses, global and regional cardiac function and on myocardial viability through the peripheral venous injection of the gadolinium-based contrast agent [26,27].

## 6. Perioperative Management of Cardiovascular Medications

Cardiovascular medications should be patient tailored considering clinical characteristics and surgical risk [2]. As a general rule, cardiovascular medications, if appropriate, effective and not contraindicated, can be continued through surgery [2]. On the other hand, in naïve patients, specific therapy needs to be started at least 2–3 weeks before surgery in order to select appropriate drug dose titration [2]. Particular attention should be paid to the antithrombotic treatment balancing bleeding vs. thrombotic risk [1,2,13,28,29].

### 6.1. Beta-Blockers 

Beta-blockers antagonize the effect of beta-adrenergic stimuli reducing heart rate, contractility, atrioventricular conduction and myocardial oxygen demand. Furthermore, by prolonging the diastolic period, beta-blockers may increase the perfusion of ischemic areas [19, 11, 12, 24]. Thus, given the proven beneficial effects in specific cardiac diseases, namely ischemic heart diseases and chronic HF, it is wise to implement, if not contraindicated, beta-blocker therapy among selected patients with the recommendation to initiate 1–2 weeks prior to surgery in order to test tolerability and safety [1,2,4,11,12,19,30,31,32].

### 6.2. Statins

Statins are potent inhibitors of cholesterol biosynthesis with “pleiotropic” effects such as improving endothelial function, enhancing the stability of atherosclerotic plaques, decreasing oxidative stress and inflammation and inhibiting the thrombogenic response [33]. The 2014 ‘ACC/AHA Guideline on Perioperative Cardiovascular Evaluation and Management of Patients Undergoing Noncardiac Surgery’ suggest initiation of statins is appropriate in patients with indications for lipid-lowering therapy and in those scheduled for vascular surgery [2,4,33].

### 6.3. ACEI-ARBs and Other Drugs

Angiotensin-converting enzyme inhibitors (ACEI), angiotensin receptor blockers (ARBs), sacubitril-valsartan, calcium channel blockers, alfa2 agonists, nitrates and diuretics follow general rules indicating they should be continued in patients currently receiving these medications but be cautious starting them 2–3 weeks before surgery, carefully monitoring blood pressure, electrolytes and renal function [1,2,3,4,5].

## 7. Perioperative Management of Antithrombotic Therapy

In patients scheduled for elective non-cardiac surgery, it is mandatory to comprehensively assess the patient’s atherothrombotic/bleeding risk in order to choose the most suitable perioperative approach regarding antiplatelet and/or anticoagulant therapy [1,2,13,29].

### 7.1. Antiplatelet Therapy

#### Aspirin

Aspirin (ASA), oral irreversible of cyclooxygenase 1 inhibitor, does not need to be discontinued in patients currently taking this medication as secondary prevention except in very high bleeding risk surgery such as transurethral resection of the prostate (TURP), neurosurgery or posterior chamber of the eye ophthalmological interventions [2,13]. If this is the case, it does need to be stopped 5 days before surgery. However, it may be resumed 24–72 h after surgery depending on the patient’s hemostasis state [2,13]. As a note, ASA should not be initiated before surgery in naïve patients [1]. It should be highlighted continuation of low dose aspirin decreases the stent thrombosis risk even when clopidogrel, prasugrel or ticagrelor are discontinued in perioperative period [1,2].

### 7.2. Dual Antiplatelet Therapy

Given the synergistic importance of the adenosine diphosphate P2Y12 and the thromboxane pathways in amplifying platelet activation, DAPT, aspirin in combination with a P2Y12 inhibitors (clopidogrel, prasugrel and ticagrelor) is the cornerstone of treatment after coronary stent implantation [11,12,34].

When approaching perioperative management of antithrombotic therapy in patients treated with coronary stents undergoing non-cardiac surgical interventions it is crucial to define the thrombotic risk of the patient vs. the bleeding risk of the procedure (Supplementary Table S2) [2,13]. It should be pointed out the thrombotic risk of the patient results from the interplay of multiple factors: patient clinical characteristics (i.e., history of CAD along with the presence of co-morbidities such as diabetes, renal dysfunction, chronic HF, peripheral artery diseases), coronary anatomy status (multiple/long bifurcation lesions, small vessels, left main involvement), type (BMS, DES, bioresorbable vascular scaffold), size and number of stents implanted, time interval from PCI to surgery along with potential adverse events of early DAPT cessation [13,35]. 

However, it is advisable to use the “Stent and Surgery app” (https://itunes.apple.com/us/app/stent-surgery/id551350096?mt.8) in order to rapidly and objectively assess the thrombotic risk of the patient vs. the hemorrhagic risk of the surgery and, consequently, choose the most appropriate management [13] (Table 3).

### 7.3. Intravenous Reversible Antiplatelet Agents 

The reversible intravenous antiplatelet agents available for clinical use, and thus of potential utility for bridging, include P2Y12 inhibitors such as cangrelor and glycoprotein IIb/IIIa inhibitors (GPIIb-IIIa) such as tirofiban and eptifibatide. In this regard, while non-deferrable surgery at low risk of bleeding can be undertaken on DAPT therapy, high bleeding risk surgery should be deferred (if possible) among patients on DAPT deemed at high risk for stent thrombosis. If not deferrable, bridging therapy with intravenous reversible antiplatelet agents may be a valuable option. If this is the case, ASA should be continued (except in the aforementioned very high bleeding risk surgery) while oral P2Y12 inhibitors should be discontinued (clopidogrel and prasugrel 5 days before and ticagrelor 7 days before surgery) with intravenous reversible antiplatelet agents bridging therapy started 2–4 days after discontinuation. Of note, cangrelor needs to be continued until 1–6 h before surgery and GPIIb-IIIa inhibitors until 4–6 h before surgery [13,36,37]. Then, patients should resume oral P2Y12 inhibitors (clopidogrel preferred) within 24–48 h after surgery, with loading dose [13].

### 7.4. Anticoagulant Therapy

The perioperative management of anticoagulant therapy remains challenging, mainly depending on the patients clinical characteristics (age, body weight, history of gastrointestinal or intracranial bleeding, renal and hepatic function, drugs interactions), the bleeding risk of surgery (minor, low and high risk) and the class (type) of anticoagulant used (vitamin K antagonists (VKAs), non-vitamin K antagonist oral anticoagulants (NOACs), heparin) [1,29].

#### 7.4.1. Vitamin K Antagonists

VKAs are a class of oral anticoagulant drugs that act as antagonists to vitamin K. The mechanism of action is to interfere with the interaction between vitamin K and coagulation factors II, VII, IX and X [38]. 

Patients on VKAs scheduled for minor bleeding risk elective surgery (i.e., dental, cataract or glaucoma interventions, endoscopy without biopsy or resection, minor skin surgery) do not need to stop anticoagulant therapy. However, it is wise to keep international normalized ratio (INR) levels in the lower therapeutic range [1]. On the other hand, patients on VKAs scheduled for low (i.e., endoscopy with biopsy, prostate or bladder biopsy, electrophysiological study or catheter ablation, pacemaker implantation, etc.) or high-risk (i.e., thoracic surgery, abdominal surgery, major orthopaedic surgery, liver and kidney biopsy, polypectomy, etc.) bleeding elective surgery do need to discontinue VKAs 3–5 days before surgery. In this regard, surgery can be safely performed when INR is ≤1.5. However, in VKAs patients with a high thromboembolic risk of (i.e., AF with a CHA2DS2 -VASc ≥4, mechanical prosthetic heart valves, newly inserted biological prosthetic heart valves or mitral valvular repair (within the past 3 months), recent venous thromboembolism (within 3 months), thrombophilia), discontinuation of VKAs should be followed by bridging therapy with a therapeutic dose of unfractionated heparin (UFH) or low molecular weight heparin (LMWH) [1]. VKAs can be resumed on day 1 or 2 after surgery depending on adequate hemostasis. It should be underlined that the use of VKAs in patients with advanced liver disease and coagulopathy is challenging due to intrinsically elevated INR values [1] (Table 4).

#### 7.4.2. Non-Vitamin K Antagonist Oral Anticoagulants

NOACs (Table 4) include apixaban, dabigatran, rivaroxaban and edoxaban. Apixaban, edoxaban and rivaroxaban inhibit Factor Xa, whereas dabigatran is a direct thrombin inhibitor [38]. All NOACs have a predictable effect (onset and offset) without need for regular anticoagulation monitoring [38]. The ‘2021 European Heart Rhythm Association Practical Guide on the Use of NOACs in Patients with Atrial Fibrillation’ recommends: (a) not to interrupt NOACs before minor bleeding risk surgery that can be performed 12–24 h after the last NOACs intake [29]; (b) to take the last dose of apixaban, edoxaban or rivaroxaban 24–36 h before low bleeding risk surgery and 24–48 h before if dabigatran is chosen [29]; and (c) to take the last dose of apixaban, edoxaban or rivaroxaban at least 48 h before high bleeding risk surgery and 48–96 h before if dabigatran is chosen [26]. Full dose of NOACs may be resumed 24 h post low-bleeding risk interventions and 48–72 h post high-bleeding risk interventions depending on hemostasis state [29]. However, as a general rule, the dose of NOACs is highly dependent on renal function along with the patient’s age (<80 vs. ≥80 years) and weight (>60 vs. ≤60 kg) [29]. It has to be highlighted that preoperative bridging with LMWH or UFH is not needed [29].

#### 7.4.3. Unfractionated Heparin and Low-Molecular-Weight Heparin

aPTT, Activated partial thromboplastin time; Da, Dalton; GFR, glomerular filtration rate; HIT, heparin-induced thrombocytopenia; LMWH, low-molecular-weight heparin; UFH, unfractionated heparin.

Heparin is a heterogeneous mixture of polysaccharide fragments with varying molecular weights: UFH and LMWH [24]. Heparin clinical use is prophylactic as well as therapeutic [24] (Table 5).

Prophylactic use is indicated for prevention of venous thromboembolism in the case of surgery carrying a moderate-high thrombotic risk (i.e., orthopaedic surgery or general surgery) or in a non-surgical setting for patients who are immobilized due to acute/chronic diseases (i.e., HF, respiratory failure, infections, etc.). In most cases, for prophylaxis, LMWH is often used in once daily subcutaneous doses of 4000–5000 UI (or 2500 to 3000 units twice daily). Alternatively, UFH may be given subcutaneously in fixed doses of 5000 UI two to three times daily. It has to be underlined that prophylactic doses of LMWH as well as UFH do not need coagulation monitoring [24].

Therapeutic use, in the preoperative setting, is indicated as “bridging therapy” in patients who require interruption of VKAs, as discussed above. For therapeutic purposes UFH is administered as an initial bolus of 5000 U followed by 30,000 to 35,000 U/24 h with activated partial thromboplastin time (aPTT) or anti factor Xa level monitoring while LMWH is used in doses of 100 U/kg twice daily (no monitoring is needed) and halved in patients with renal impairment (Creatinine Clearance (ClCr) < 30 mL/min) [24].

In general, due to efficacy/safety better evidence, LMWH as “bridging therapy” is preferred to UFH except in patients with mechanical prosthetic heart valves. As a note, UFH should be discontinued 4 h before surgery and low molecular weight heparin 12 h before and then resumed 12 h after the procedure [1].

It should be highlighted that protamine sulphate can be utilized to neutralize heparin in serious bleeding, most often occurring in cases of concomitant use of antiplatelet/fibrinolytic drugs and/or recent surgery or trauma. Other side effects include, especially for UFH, heparin-induced thrombocytopenia (HIT), elevated levels of transaminases and osteoporosis [24].

## 8. Post-Operative Major Adverse Cardiovascular Events Prevention

During the high-risk patient’s post-operative phase, it remains essential to prevent MACEs. In this regard, the caring team should pay attention to pain, oxygenation and hemodynamics control. Levels of hemoglobin and hematocrit should be maintained >10 and >30, respectively. Furthermore, intake should be within 500 cc output in order to avoid preload increase and heart failure. In case of rapid change of symptoms and/or hemodynamic instability, cardiologist consult along with ECG, biomarkers testing (namely natriuretic peptides and troponin) and FoCUS exam is warranted to ascertain potential cardiovascular causes and rapidly implement appropriate diagnostic-therapeutic strategies [3,4].

## 9. Conclusions

MACEs are common causes of perioperative mortality and major morbidity. Minimizing these complications requires a careful preoperative integrated multidisciplinary assessment including definition of surgical risk per se and related timing exclusion of clinical unstable conditions or recent coronary revascularization estimation of functional capacity and overall cardiovascular perioperative risk (Figure 1). The implementation of cardiovascular non-invasive/invasive tests and therapeutic interventions are dependent on patient and surgery specific characteristics (personalized medicine model). Finally, during the COVID-19 pandemic, careful attention should be paid to minimize the risk of infection transmission when performing cardiac consultation.

## Figures and Tables

**Table 1:** Measures to prevent SARS-CoV-2 exposure and transmission in the setting of preoperative assessment and management of cardiovascular risk among patients undergoing non-cardiac surgery [6,7].

**General Recommendations**
• At the time of hospital admission, patients should undergo clinical and laboratory assessment for COVID-19 infection + SARS-CoV-2 nucleic acid amplification testing (for example, RT-PCR) or antigen testing.
• Use “*personal protective equipment*” (protective masks/clothing, gloves, googles, surgical cap, etc.).
• Observe social distancing rules and routinely clean medical equipment.
• Implement teleconsulting/telemedicine whenever possible and limit number of caregivers.
• Patients should undergo SARS-CoV-2 polymerase chain reaction 48–72 h before invasive/high-risk contamination procedures (i.e., coronary angiography, TEE, physical stress tests, etc.).
• Perform periodic COVID-19 screening of patients and caregivers (SARS-CoV-2 nucleic acid amplification testing (for example, RT-PCR) or antigen testing.
• Check COVID-19 vaccination status for patients and caregivers.
**Cardiac Imaging**
• Cardiac imaging should be performed if appropriate and only if it is likely to substantially change patient management.
• A focused cardiac ultrasound study (FoCUS) is recommended to reduce the duration of exposure. Handheld ultrasound devices should be implemented.
• The risk of contamination of equipment and personnel is very high during TEE; if necessary, consider as alternatives CCTA or cardiac CMRI.
• CCTA may be implemented to exclude or confirm coronary heart disease.
• Avoid cardiac stress tests; if necessary, favor pharmacologic (or CCTA) to physical stress.
• If an urgent coronary angiography is needed (i.e., ACS), a dedicated *COVID-19 Cath-Lab* should be used.
• CMRI may be implemented in the suspicion of takotsubo syndrome or myocarditis.

ACS, acute coronary syndrome; cath lab, cardiac catheterization laboratory; CCTA, coronary computed tomographic angiography; CMRI, cardiac magnetic resonance imaging; COVID-19, 2019 Coronavirus disease; RT-PCR, reverse transcription polymerase chain reaction; SARS-CoV-2, severe acute respiratory syndrome coronavirus-2; TEE, transesophageal echocardiogram.

**Table 2:** Assessment of functional capacity [18].

Activities	Wheight (METs)
• Can you take care of yourself (eating, dressing, bathing or using the toilet)?	2.75
• Can you walk indoors, such as around your house?	1.75
• Can you walk a block or two on level ground?	2.75
• Can you climb a flight of stairs or walk up a hill?	5.50
• Can you run a short distance?	8.00
• Can you undertake light work around the house, such as dusting or washing dishes?	2.70
• Can you perform moderate work around the house, such as vacuuming, sweeping floors or carrying in groceries?	3.50
• Can you perform heavy work around the house, such as scrubbing floors or lifting and moving heavy furniture?	8.00
• Can you perform yard work, such as raking leaves, weeding or pushing a power mower?	4.50
• Can you have sexual relations?	5.25
• Can you participate in moderate recreational activities, such as golf, bowling, dancing, doubles tennis or throwing a baseball or football?	6.00
• Can you participate in strenuous sports, such as swimming, singles tennis, football, basketball or skiing?	7.50

METs, metabolic equivalents.

**Table 3:** Websites/Apps of interest [1,2,3,4,6,7,29].

Ref/Web-Sites/Apps	Link
2014 ‘ESC/ESA Guidelines on non-cardiac surgery: Cardiovascular assessment and management’.	https://www.escardio.org/Guidelines/Clinical-Practice-Guidelines/ESC-ESA-Guidelines-on-non-cardiac-surgery-cardiovascular-assessment-and-managem
‘ACC/AHA Guideline on Perioperative Cardiovascular Evaluation and Management of Patients Undergoing Noncardiac Surgery’.	https://www.ahajournals.org/doi/pdf/10.1161/CIR.0000000000000106
‘CCS Guidelines on Perioperative Cardiac Risk Assessment and Management for Patients Who Undergo Noncardiac Surgery’.	https://www.onlinecjc.ca/article/S0828-282X(16)30980-1/pdf
2021 ‘EHRA Practical Guide on the Use of Non-Vitamin K Antagonist Oral Anticoagulants in Patients with Atrial Fibrillation’.	https://www.escardio.org/Guidelines/Recommended-Reading/Heart-Rhythm/Novel-Oral-Anticoagulants-for-Atrial-Fibrillation
‘ESC Guidance for the Diagnosis and Management of CV Disease during the COVID-19 Pandemic’.	https://www.escardio.org/static-file/Escardio/Education-General/Topic%20pages/Covid-19/ESC%20Guidance%20Document/ESC-Guidance-COVID-19-Pandemic.pdf
‘COVID-19 pandemic and cardiac imaging: EACVI recommendations on precautions, indications, prioritization, and pro-tection for patients and healthcare personnel’.	https://academic.oup.com/ehjcimaging/article/21/6/592/5815408
Stent and Surgery App	https://play.google.com/store/apps/details?id=com.araneum.stentsurgery&hl=it&gl=US
MDCalc Medical Calculator App	https://play.google.com/store/apps/details?id=com.mdaware.mdcalc&hl=it&gl=US • ASA Physical Status/ASA Classification. • Creatinine clearance (Cockcroft-Gault Equation). • CHA_2_DS_2_-VASc Score. • Child–Pugh Score. • QT corrected interval (QTc). • HAS-BLED Score. • Revised Cardiac Risk Index.

ACC, American College of Cardiology; AHA, American Heart Association; CCS, Canadian Cardiovascular Society; EACVI, European Association of Cardiovascular Imaging; EHRA, European Heart Rhythm Association; ESA, European Society of Anesthesiology; ESC, European Society of Cardiology.

**Table 4:** Management of VKAs and NOACs in patients undergoing non-cardiac surgery [1,29].

**VKAs**	➢ **Elective surgery**
	• Minor bleeding risk surgery: no change in oral anticoagulation therapy is needed but keep INR levels in the lower therapeutic range.
	• Low/high bleeding risk surgery: take the last dose 3–5 days before surgery; when INR is ≤1.5, surgery can be performed safely.
	➢ VKAs should be resumed on day 1 or 2 after surgery depending on the patient’s hemostatic status, but no less than 12 h after the procedure. ➢ *If high risk of thromboembolism* ^1^*, bridging therapy with UFH or LMWH* ^2^ *needs to be implemented*. ➢ In case of urgent/emergent surgery, immediately discontinue. If needed, reversal with vitamin K or fresh-frozen plasma/PCCs.
**NOACs**	➢ **Elective surgery**
	Take the last dose before surgery according to CrCl:
	• Minor bleeding risk surgery: ≥12 h or 24 h ^1^ • Low bleeding risk surgery: - apixaban/edoxaban/rivaroxaban ≥24 h ^2^ - dabigatran ≥ 24–48 h ^3^
	• High bleeding risk surgery: - apixaban/edoxaban/rivaroxaban ≥48 h ^4^ - dabigatran ≥48–96 h ^5^
	➢ *No bridging with heparin required*. ➢ In case of urgent/emergent surgery immediately discontinue. If needed, reversal with idaracizumab (dabigatran) or PCCs or aPCCs.

aPCCs, activated prothrombin complex concentrate; CrCL, creatinine clearance; INR, international normalized ratio; LMWH, low molecular weight heparin; NOACs, non-vitamin K antagonist oral anticoagulants; PCCs, prothrombin complex concentrate; UFH, unfractionated heparin; VKAs, vitamin K antagonists. ^1^ Patients with at least one of the following: AF with a CHA2DS2-VASc score ≥ 4, mechanical prosthetic heart valves, newly inserted biological prosthetic heart valves, mitral valvular repair (within the past 3 months), recent venous thromboembolism (within 3 months), thrombophilia. ^2^ CrCl > 30 mL/min: ≥24 h; CrCl < 29 mL/min: ≥36 h; CrCl < 15 mL/min: not indicated. ^3^ CrCl ≥ 80 mL/min: ≥24 h; CrCl 50–79 mL/min: ≥36 h; CrCl 30–49 mL/min: ≥48; CrCl < 29 mL/min: not indicated. ^4^ CrCl > 15 mL/min: ≥48 h; CrCl < 15 mL/min: not indicated. ^5^ CrCl ≥ 80 mL/min: ≥48 h; CrCl 50–79 mL/min: ≥72 h; CrCl 30–49 mL/min: ≥96; CrCl < 29 mL/min: not indicated.

**Table 5:** Characteristics of unfractionated heparin (UFH) vs. low molecular weight heparin (LMWH) and their management in patients undergoing non-cardiac surgery [1,24].

Features	UFH	LMWH
Mean molecular weight	15,000 Da	5000 Da
Target	Xa and IIa	Xa and IIa (greater Xa inhibition than IIa)
Bioavailability (%)	30	90
Half-life	1 h	4 h
Renal Excretion	No	Yes
Antidote (Protamine sulfate)	Complete reversal	Partial reversal (~50%)
Heparin-induced thrombocytopenia (HIT)	<5%	<1%
Method of administration	Intravenous infusion or less frequently subcutaneously.	Subcutaneously (less frequently can be administered intravenously if a rapid anticoagulant response is needed).
Monitoring	aPTT	Not necessary (predictable anticoagulant response).
Dosages • Prophylaxis • Therapeutic	- Usually given in fixed doses of 5000 units subcutaneously two or three times daily. *	- 4000 to 5000 units daily or 2500 to 3000 units twice daily subcutaneously.
	- Initial bolus of 5000 U followed by 30,000 to 35,000 U/24 h followed by intravenous infusion with aPTT monitoring.	- Subcutaneously according to body weight (100 U/kg twice daily). - The dose needs to be reduced in patients with renal impairment (GFR < 30 mL/min/1.73 m^2^).
Management before non-cardiac surgery	- Discontinue administration ≥4 h before surgery. - Resume full dose ≥12 h after surgery. - In case of urgent/emergent surgery immediately discontinue. If needed, complete reversal with protamine sulphate.	- Discontinue administration ≥12 h before surgery. - Resume full dose ≥12 h after surgery. - In case of urgent/emergent surgery immediately discontinue. If needed, partial reversal (~50%) with protamine sulphate.
Limitations	Dose-dependent clearance (binds to endothelial cells); variable anticoagulant response (binds to plasma proteins).	Potential accumulation in patients with renal insufficiency (GFR <30 mL/min/1.73 m^2^).
Side effects	- *Short term:* • bleeding (most common, increasing with higher heparin doses or concomitant administration of antiplatelet or fibrinolytic agents); • HIT (it occurs 5 to 14 days after the initiation of heparin therapy, but it may be manifested earlier if the patient has received heparin within the past 3 months); • elevated levels of transaminases (rapidly return to normal when the drug is stopped). - *Long term:* • osteoporosis.	The same as UFH but less frequent.

* Monitoring of coagulation is unnecessary.

## Data Availability

The data that support the findings of this review are openly available in the References section.

## Supplementary Materials

The following are available online at https://www.mdpi.com/article/10.3390/jcdd8100126/s1, Table S1: Classification of elective surgical interventions according to the surgical risk of the procedures, Table S2: Classification of elective surgical interventions according to bleeding risk of surgical and anesthesiologic procedures.

## Abbreviations

AF	atrial fibrillation
ARBs	angiotensin receptor blockers
ASA	aspirin
BMS	bare metal stent
BNP	brain natriuretic peptide
CA	coronary angiography
CABG	coronary artery bypass grafting
CAD	coronary artery disease
CCTA	coronary computed tomographic angiography
CMRI	cardiac magnetic resonance imaging
COVID-19	2019 Coronavirus disease
ClCr	creatinine clearance
CT	computed tomography
DAPT	dual antiplatelet therapy
DES	drug eluting stent
ECG	electrocardiogram
FoCUS	focused cardiac ultrasound study
GFR	glomerular filtration rate
GPIIb-IIIa	glycoprotein IIb/IIIa inhibitors
HF	heart failure
INR	international normalized ratio
LMWH	low molecular weight heparin
MACEs	major adverse cardiac events
METs	metabolic equivalents
MI	myocardial infarction
NOACs	non-vitamin K antagonist oral anticoagulants
NSQIP	National Surgical Quality Improvement Program
NT-proBNP	N-terminal-pro hormone BNP
PCI	percutaneous coronary intervention
RCRI	Revised Cardiac Risk Index
SARS-CoV-2	severe acute respiratory syndrome coronavirus-2
SPECT	single-photon emission computed tomography
TTE	transthoracic color-Doppler echocardiography
TURP	transurethral resection of the prostate
UFH	unfractionated heparin
VKAs	vitamin K antagonists

## References

[1] Kristensen S.D., Knuuti J., Saraste A., Anker S.D., Bøtker H.E., De Hert S., Ford I., Juanatey J.R.G., Gorenek B., Heyndrickx G.R. (2014). 2014 ESC/ESA Guidelines on non-cardiac surgery: Cardiovascular assessment and management. Eur. Heart J..

[2] Smilowitz N.R., Berger J.S. (2020). Perioperative Cardiovascular Risk Assessment and Management for Noncardiac Surgery. JAMA.

[3] Duceppe E., Parlow J., MacDonald P., Lyons K., McMullen M., Srinathan S., Graham M., Tandon V., Styles K., Bessissow A. (2016). Canadian Cardiovascular Society Guidelines on Perioperative Cardiac Risk Assessment and Management for Patients Who Undergo Noncardiac Surgery. Can. J. Cardiol..

[4] Fleisher L.A., Fleischmann K.E., Auerbach A., Barnason S.A., Beckman J., Bozkurt B., Davila-Roman V.G., Gerhard-Herman M.D., Holly T.A., Kane G.C. (2014). 2014 ACC/AHA Guideline on Perioperative Cardiovascular Evaluation and Management of Patients Undergoing Noncardiac Surgery. Circulation.

[5] Zipes D.P., Libby P., Bonow R.O., Mann D.L., Tomaselli G.F. (2018). Braunwald’s Heart Disease: A Textbook of Cardiovascular Medicine.

[6] Skulstad H., Cosyns B., Popescu B.A., Galderisi M., Di Salvo G., Donal E., Petersen S., Gimelli A., Haugaa K., Muraru D. (2020). COVID-19 pandemic and cardiac imaging: EACVI recommendations on precautions, indications, prioritization, and protection for patients and healthcare personnel. Eur. Heart J. Cardiovasc. Imaging.

[7] The European Society for Cardiology (2020). ESC Guidance for the Diagnosis and Management of CV Disease during the COVID-19 Pandemic. https://www.escardio.org/Education/COVID-19-and-Cardiology/ESC-COVID-19-Guidance.

[8] Bossone E., Mauro C., Maiellaro A., Raiola E., Cocchia R., Ranieri B., Sepe C., Capone V., Chianese S., Maramaldi R. (2021). Cardiac teleconsulting in the time of COVID-19 global pandemic: The “Antonio Cardarelli” Hospital project. Monaldi Arch. Chest Dis..

[9] Società Italiana di Ecocardiografia e Cardiovascular Imaging (SIECVI) (2020). Documento ad uso Degli Operatori di Ecografia Cardiovascolare per COVID-19. https://www.siec.it/download/siecvi-documento-ad-uso-degli-operatori-di-ecografia-cardiovascolare-per-covid-19.

[10] Giustino G., Chieffo A., Palmerini T., Valgimigli M., Feres F., Abizaid A., Costa R.A., Hong M.-K., Kim B.-K., Jang Y. (2016). Efficacy and Safety of Dual Antiplatelet Therapy After Complex PCI. J. Am. Coll. Cardiol..

[11] Ibanez B., James S., Agewall S., Antunes M.J., Bucciarelli-Ducci C., Bueno H., Caforio A.L.P., Crea F., Goudevenos J.A., Halvorsen S. (2017). 2017 ESC Guidelines for the management of acute myocardial infarction in patients presenting with ST-segment elevation. Eur. Heart J..

[12] Collet J.-P., Thiele H., Barbato E., Barthélémy O., Bauersachs J., Bhatt D.L., Dendale P., Dorobantu M., Edvardsen T., Folliguet T. (2020). 2020 ESC Guidelines for the management of acute coronary syndromes in patients presenting without persistent ST-segment elevation. Eur. Heart J..

[13] Rossini R., Tarantini G., Musumeci G., Masiero G., Barbato E., Calabrò P., Capodanno D., Leonardi S., Lettino M., Limbruno U. (2018). A Multidisciplinary Approach on the Perioperative Antithrombotic Management of Patients with Coronary Stents Undergoing Surgery. JACC: Cardiovasc. Interv..

[14] Lee T.H., Marcantonio E.R., Mangione C.M., Thomas E.J., Polanczyk C.A., Cook E.F., Sugarbaker D.J., Donaldson M.C., Poss R., Ho K.K.L. (1999). Derivation and Prospective Validation of a Simple Index for Prediction of Cardiac Risk of Major Noncardiac Surgery. Circulation.

[15] Gupta P.K., Gupta H., Sundaram A., Kaushik M., Fang X., Miller W.J., Esterbrooks D.J., Hunter C.B., Pipinos I.I., Johanning J.M. (2011). Development and Validation of a Risk Calculator for Prediction of Cardiac Risk After Surgery. Circulation.

[16] Fletcher G.F., Balady G.J., Amsterdam E.A., Chaitman B., Eckel R., Fleg J., Froelicher V.F., Leon A.S., Piña I.L., Rodney R. (2001). Exercise Standards for Testing and Training. Circulation.

[17] Myers J., Prakash M., Froelicher V., Do D., Partington S., Atwood J.E. (2002). Exercise Capacity and Mortality among Men Referred for Exercise Testing. New Engl. J. Med..

[18] Hlatky M., Boineau R.E., Higginbotham M.B., Lee K.L., Mark D., Califf R.M., Cobb F.R., Pryor D.B. (1989). A brief self-administered questionnaire to determine functional capacity (The Duke Activity Status Index). Am. J. Cardiol..

[19] Ponikowski P., Voors A.A., Anker S.D., Bueno H., Cleland J.G.F., Coats A.J.S., Falk V., González-Juanatey J.R., Harjola V.-P., Jankowska E.A. (2016). 2016 ESC Guidelines for the diagnosis and treatment of acute and chronic heart failure. Eur. Heart J..

[20] Agewall S., Giannitsis E., Jernberg T., Katus H. (2010). Troponin elevation in coronary vs. non-coronary disease. Eur. Heart J..

[21] Feigenbaum H., Armstrong W.F., Ryan T. (2019). Feigenbaum’s Echocardiography.

[22] Cardim N., Dalen H., Voigt J.-U., Ionescu A., Price S., Neskovic A.N., Edvardsen T., Galderisi M., Sicari R., Donal E. (2018). The use of handheld ultrasound devices: A position statement of the European Association of Cardiovascular Imaging (2018 update). Eur. Heart J. Cardiovasc. Imaging.

[23] Knuuti J., Wijns W., Saraste A., Capodanno D., Barbato E., Funck-Brentano C., Prescott E., Storey R., Deaton C., Cuisset T. (2019). 2019 ESC Guidelines for the diagnosis and management of chronic coronary syndromes. Eur. Heart J..

[24] Zipes D.P., Libby P. (2019). Braunwald’s Heart Disease: A Textbook of Cardiovascular Medicine, 2-Volume Set.

[25] Serruys P.W., Hara H., Garg S., Kawashima H., Nørgaard B.L., Dweck M.R., Bax J.J., Knuuti J., Nieman K., Leipsic J.A. (2021). Coronary Computed Tomographic Angiography for Complete Assessment of Coronary Artery Disease. J. Am. Coll. Cardiol..

[26] Lombardi M., Plein S. (2018). The EACVI Textbook of Cardiovascular Magnetic Resonance.

[27] Hundley W.G., Bluemke D.A., Finn J.P., Flamm S.D., Fogel M.A., Friedrich M.G., Ho V.B., Jerosch-Herold M., Kramer C.M., Manning W.J. (2010). ACCF/ACR/AHA/NASCI/SCMR 2010 expert consensus document on cardiovascular magnetic resonance: A report of the American College of Cardiology Foundation Task Force on Expert Consensus Documents. J. Am. Coll. Cardiol..

[28] Levine G.N., Bates E.R. (2016). 2016 ACC/AHA Guideline Focused Update on Duration of Dual Antiplatelet Therapy in Patients with Coronary Artery Disease. Circulation.

[29] Steffel J., Collins R., Antz M., Cornu P., Desteghe L., Haeusler K.G., Oldgren J., Reinecke H., Roldan-Schilling V., Rowell N. (2021). 2021 European Heart Rhythm Association Practical Guide on the Use of Non-Vitamin K Antagonist Oral Anticoagulants in Patients with Atrial Fibrillation. EP Eur..

[30] London M.J., Hur K., Schwartz G.G., Henderson W.G. (2013). Association of Perioperative β-Blockade with Mortality and Cardiovascular Morbidity Following Major Noncardiac Surgery. JAMA.

[31] Flu W.-J., Van Kuijk J.-P., Chonchol M., Winkel T.A., Verhagen H.J., Bax J.J., Poldermans D. (2010). Timing of Pre-Operative Beta-Blocker Treatment in Vascular Surgery Patients: Influence on Post-Operative Outcome. J. Am. Coll. Cardiol..

[32] Wijeysundera D., Duncan D., Nkonde-Price C., Virani S., Washam J.B., Fleischmann K.E., Fleisher L.A. (2014). Perioperative Beta Blockade in Noncardiac Surgery: A Systematic Review for the 2014 ACC/AHA Guideline on Perioperative Cardiovascular Evaluation and Management of Patients Undergoing Noncardiac Surgery. J. Am. Coll. Cardiol..

[33] Mach F., Baigent C., Catapano A.L., Koskinas K.C., Casula M., Badimon L., Chapman M.J., De Backer G.G., Delgado V., Ference B.A. (2019). 2019 ESC/EAS Guidelines for the management of dyslipidaemias: Lipid modification to reduce cardiovascular risk. Eur. Heart J..

[34] Gremmel T., Yanachkov I., Yanachkova M.I., Wright G.E., Wider J., Undyala V.V., Michelson A.D., FrelingerIII A.L., Przyklenk K. (2016). Synergistic Inhibition of Both P2Y 1 and P2Y 12 Adenosine Diphosphate Receptors as Novel Approach to Rapidly Attenuate Platelet-Mediated Thrombosis. Arter. Thromb. Vasc. Biol..

[35] Eisen A., Bhatt D.L. (2015). Defining the optimal duration of DAPT after PCI with DES. Nat. Rev. Cardiol..

[36] Rossini R., Musumeci G., Visconti L.O., Bramucci E., Castiglioni B., De Servi S., Lettieri C., Lettino M., Piccaluga E., Savonitto S. (2014). Perioperative management of antiplatelet therapy in patients with coronary stents undergoing cardiac and non-cardiac surgery: A consensus document from Italian cardiological, surgical and anaesthesiological societies. EuroIntervention.

[37] Angiolillo D.J., Schneider D.J., Bhatt D.L., French W.J., Price M.J., Saucedo J.F., Shaburishvili T., Huber K., Prats J., Liu T. (2012). Pharmacodynamic effects of cangrelor and clopidogrel: The platelet function substudy from the cangrelor versus standard therapy to achieve optimal management of platelet inhibition (CHAMPION) trials. J. Thromb. Thrombolysis.

[38] Mekaj A., Mekaj Y., Duci S., Miftari E. (2015). New oral anticoagulants: Their advantages and disadvantages compared with vitamin K antagonists in the prevention and treatment of patients with thromboembolic events. Ther. Clin. Risk Manag..

