# Supplementary material for: Preoperative Assessment and Management of Cardiovascular Risk in Patients Undergoing Non-Cardiac Surgery: Implementing a Systematic Stepwise Approach during the COVID-19 Pandemic Era
Source: J Cardiovasc Dev Dis. 2021 Oct 3;8(10):126. doi: 10.3390/jcdd8100126 (PMC8541645; doi:10.3390/jcdd8100126)
Supplement: Supplementary file 1 [file jcdd-08-00126-s001.zip › jcdd-1372034-suppl.pdf]

Table S1. Classification of elective surgical interventions according to the surgical risk of the procedures.

Surgical interventions	Low Risk	Moderate Risk	High Risk
**Urology**	• Flexible cystoscopy<br>• Ureteral catheterization<br>• Scrotal and penile surgery<br>• Percutaneous lithotripsy<br>• TURP, TURBT, endoscopic bladder/prostatic interventions<br>• Ureteroscopy	• Radical and partial nephrectomy<br>• Percutaneous nephrostomy<br>• Percutaneous lithotripsy<br>• Radical prostatectomy<br>• TURP, TURBT, endoscopic bladder/prostatic interventions	• Radical cystectomy
**Digestive endoscopy**	• EGDS or colonoscopy +/- biopsy<br>• Echoendoscopy without biopsy<br>• Polypectomy polyps (> or <1cm)<br>• ERCP, stent, papillae dilation without sphincterotomy<br>• Endoscopy + FNA for solid lesions<br>• Stenosis dilatation (oesophageal, colorectal)<br>• Gastroenteric stents<br>• Argon plasma coagulation treatment<br>• PEG<br>• Oesophageal variceal ligation/sclerosis<br>• Haemorrhoid ligation/sclerosis<br>• Dilation due to achalasia<br>• Mucosectomy/submucosal resection<br>• Ultrasound with FNA biopsy of pancreatic cystic lesions<br>• Vater papilla ampullectomy		
**General surgery**	• Hernioplasty, plastic surgery of incisional hernias<br>• Breast surgery, Superficial biopsies, lymph node biopsies<br>• Incision of abscesses<br>• Thyroidectomy<br>• Hemorrhoidectomy	• Appendectomy, colectomy, gastric resection, intestinal resections, cholecystectomy<br>• Gastrectomy, splenectomy<br>• Obesity surgery, rectal resection, cholecystectomy	• Esophagectomy, pancreaticoduodenectomy, liver resections, biliary tract surgery, repair of perforated bowel<br>• Adrenal resection<br>• Liver transplant
**Orthopaedic surgery**	• Hand and foot surgery (trigger finger, hammer toe, hallux valgus, M. Dupuytren)<br>• Shoulder and knee arthroscopy	• Demolition and reconstructive cancer surgery of the head and neck area (laryngectomy)<br>• Shoulder prosthetic surgery<br>• Spine surgery<br>• Knee surgery (ACL osteotomies)<br>• Major prosthetic surgery (hip, knee)<br>• Major traumatology (pelvis, long bones)<br>• Fractures of the proximal femur in the elderly	
**Thoracic surgery**		• Diagnostic videothoracoscopy, Chest wall lesions resection	• Lobectomy (Wedge, typical), pneumonectomy<br>• Mediastinal mass excision

			• Pleuropneumonectomy<br>• Decortication of the lung
**Vascular surgery**		• Carotid endarterectomy, PTA, EVAR	• Open aortic surgery<br>• Open revascularization or lower limb amputation
**Ophtalmology**	• Vitrectomy, trabeculectomy<br>• Cataract or glaucoma correction surgery<br>• Intravitreal injections		
**Gynaecology**	• Diagnostic hysteroscopy<br>• Resectoscopic hysteroscopy polypectomy, metroplasty<br>• Dilatation and curettage of the uterine cavity<br>• Conization with diathermic loop of the portio<br>• Marsupialization/removal of Bartholin's gland cyst<br>• Diagnostic or operative minimal laparoscopy (simple adhesiolysis, endometriotic implants)	• Resectoscopic hysteroscopy myomectomy, endometrial ablation<br>• Laparoscopic/laparotomic surgery for endometriosis<br>• Laparoscopic/laparotomic excision of appendages for benign pathology<br>• Simple abdominal hysterectomy for benign pathology<br>• Simple vaginal hysterectomy for benign pathology / prolapse<br>• Vaginal fascial reparative surgery (cystocele/rectocele repair), vaginal prosthetic reparative surgery, vulvar radical surgery<br>• Omentectomy<br>• Laparoscopic/laparotomic hysterectomy for large uteri (>750g)<br>• Laparotomic/laparoscopic myomectomy<br>• Debulking surgery for ovarian cancer<br>• Radical surgery for portio and endometrial cancer<br>• Pelvic/lumbo-aortic lymphadenectomy	• Pelvic evisceration
**Otolaryngology**	• Closed reduction of zygomatic arch fracture, closed reduction of mandibular fracture<br>• Open reduction of jaw's fracture; orthognathic surgery<br>• Tonsillectomy, adenoidectomy, FESS, parotidectomy<br>• Rhino/rhinoseptoplasty, tympanoplasty, myringoplasty<br>• Ossicular chain reconstruction<br>• Closed reduction of nasal bone fracture		
**Plastic surgery**	• Exeresis and suture of small epitheliomas and small benign skin lesions, scar correction<br>• Treatment of soft tissue pathologies of the hand	• Removal of large tumors of the soft tissues of the face and neck and plastic reconstruction using a microsurgical flap.<br>• Functional treatment of trauma outcomes (accidents roads, outcomes of surgical interventions, etc), losses of post-demolition substance of considerable entity of face, lower and upper limbs, of the abdomen, of back, by means of flaps, microsurgical or pluritissutal pedunculated transposition flaps of considerable entity.	

	• Upper blepharoplasty, lower blepharoplasty<br>• Otoplasty	Liposuction of abdomen, lower and upper limbs, of considerable entity.<br>Surgical treatment of burns<br>• Abdominoplasty<br>• Post-mastectomy breast reconstruction with microsurgical flap. Breast reduction
**Dentistry**	• Surgical periodontal therapy (resective surgery, regenerative surgery, mucogingival surgery), Oral surgery in general (extractive surgery, reconstructive surgery pre-implant), implant surgery	
**Vascular intervention procedures**	• CVC tunneled – Port positioning<br>• Caval filter extraction<br>• Angiography<br>• Trans-jugular liver biopsy<br>• Chemoembolization<br>• Uterine embolization (leiomyomas)<br>• Drainage replacement (biliary, nephrostomy, abdominal)<br>• TIPS	
**Neurosurgery**		• Spinal neurosurgery (herniated disc, laminectomy)<br>• Cranial neurosurgery: external ventricular lead, intraventricular catheter for intracranial pressure monitoring<br>• Cranial neurosurgery: ventriculoperitoneal shunt, extradural lesion removal, removal of intradural lesions (intracerebral tumors, intraparenchymal haemorrhage)
**Other intervention procedures**	• Paracentesis, thoracentesis<br>• Needle aspirations of superficial organs (thyroid, lymph nodes, salivary cells, breast)<br>• Aspirations, drainages and surface biopsies<br>• Intra-articular aspiration/injection<br>• Renal and hepatic thermo ablation<br>• Non-superficial percutaneous biopsies (excluding kidney and liver)<br>• Drainage of deep abscesses (abdominal, hepatic)<br>• Cholecystostomy, gastrostomy<br>• Percutaneous kidney and liver biopsy<br>• Percutaneous interventional urinary tract<br>• Percutaneous biliary intervention	• Submandibular sialoadenectomy

CVC, central venous catheters; EBUS, endobronchial ultrasound; EGDS, esophagogastroduodenoscopy; ERCP, endoscopic retrograde cholangiopancreatography; EVAR, endovascular aneurysm repair; FESS, functional endoscopic sinus surgery; FNA, fine needle biopsy; PEG, percutaneous endoscopic gastrostomy; PICC, peripherally inserted central catheter; PTA, percutaneous transluminal angioplasty; TIPS, trans-jugular intrahepatic porto-systemic shunt; TURBT, transurethral resection of bladder tumor; TURP, transurethral resection of prostate.

Table S2. Classification of elective surgical interventions according to bleeding risk (minor, low, high) of surgical and anaesthesiologic procedures.

Surgical interventions	Minor risk	Low risk	High risk
**Urology**	• Flexible cystoscopy	• Scrotal and penile surgery	• Radical and partial nephrectomy
	• Ureteral catheterization	• Prostate biopsy	• Percutaneous nephrostomy
	• Ureteroscopy		• Percutaneous lithotripsy
			• Radical cystectomy and prostatectomy
			• TURP, TURBT, endoscopic bladder/prostatic interventions
**Digestive endoscopy**	• EGDS or colonoscopy +/- biopsy	• Endoscopy + FNA for solid lesions	• Dilation due to achalasia
	• Echoendoscopy without biopsy	• Stenosis dilatation (oesophageal, colorectal)	• Mucosectomy/submucosal resection
	• Polypectomy polyps <1cm	• Gastroenteric stents	• Ultrasound with FNA biopsy of pancreatic cystic lesions
	• Endoscopic retrograde, ERCP, stent, papillae dilation without sphincterotomy	• Argon plasma coagulation treatment	• Vater papilla ampullectomy
		• ERCP	
		• Polypectomy polyps >1cm	
		• PEG	
		• Oesophageal variceal ligation/sclerosis	
		• Haemorrhoid ligation/sclerosis	
**General surgery**	• Hernioplasty, plastic surgery of incisional hernias	• Gastrectomy, gastric resection, splenectomy, cholecystectomy, appendectomy, intestinal resections, colectomy, rectal resection, hemorrhoidectomy	• Esophagectomy, pancreaticoduodenectomy, liver resections, biliary tract surgery
	• Incision of abscesses	• Obesity surgery	
	• Breast surgery	• Thyroidectomy	
	• Superficial biopsies, lymph node biopsies		
**Orthopaedic surgery**	• Hand and foot surgery (trigger finger, hammer toe, hallux valgus, M. Dupuytren)	• Shoulder prosthetic surgery	• Major prosthetic surgery (hip, knee)
	• Shoulder and knee arthroscopy	• Spine surgery	• Major traumatology (pelvis, long bones)
		• Knee surgery (ACL osteotomies)	• Fractures of the proximal femur in the elderly
**Thoracic surgery**	• Diagnostic videothoracoscopy	• Lobectomy (Wedge , typical), pneumonectomy	• Esophagectomy, pleuropneumonectomy
	• Chest wall lesions resection	• Mediastinoscopy	• Decortication of the lung
		• Sternotomy	
		• Mediastinal mass excision	
**Ophthalmology**	• Intravitreal injections	• Vitrectomy	• None
	• Cataract or glaucoma correction surgery	• Trabeculectomy	
	• Peribulbar anesthesia		
**Pneumology**	• Bronchoscopy without biopsy	• Bronchial biopsies	• Transbronchial lung biopsies
			• Operative bronchoscopy (with rigid bronchoscope)
**Vascular surgery**	• Angiography	• Carotid endarterectomy	• Open aortic surgery
		• PTA	• Open revascularization or lower limb amputation
		• EVAR	

Gynaecology	• Diagnostic hysteroscopy	• Resectoscopic hysteroscopy myomectomy, endometrial ablation	• Laparoscopic/laparotomic hysterectomy for large uteri (>750g)
	• Resectoscopic hysteroscopy polypectomy, metroplasty	• Laparoscopic/laparotomic surgery for endometriosis	• Laparotomic/laparoscopic myomectomy
	• Dilatation and curettage of the uterine cavity	• Laparoscopic/laparotomic excision of appendages for benign pathology	• Debulking surgery for ovarian cancer
	• Conization with diathermic loop of the portio	• Simple abdominal hysterectomy for benign pathology	• Radical surgery for portio and endometrial cancer
	• Marsupialization/removal of Bartholin's gland cyst	• Simple vaginal hysterectomy for benign pathology / prolapse	• Pelvic/lumbo-aortic lymphadenectomy
	• Diagnostic or operative minimal laparoscopy (simple adhesiolysis, endometriotic implants)	• Vaginal fascial reparative surgery (cystocele/rectocele repair), vaginal prosthetic reparative surgery, vulvar radical surgery	• Pelvic evisceration
		• Omentectomy	
Dentistry	• Non-surgical periodontal therapy (including ablation of tartar)	• Surgical periodontal therapy (resective surgery, regenerative surgery, mucogingival surgery)	• None
	• Endodontic therapy non-surgical	• Oral surgery in general (extractive surgery, reconstructive surgery pre-implant)	
	• Positioning of the rubber dam	• Implant surgery	
Otolaryngology	• Superficial biopsies (tonsil, tongue, nasopharynx)	• Tonsillectomy, adenoidectomy, mastoidectomy, parotidectomy	• Demolition and reconstructive cancer surgery of the head and neck area (laryngectomy)
	• Tympanic drainage	• FESS	
	• Direct microlaryngoscopy	• Rhino/rhinoseptoplasty, tympanoplasty, myringoplasty	
		• Ossicular chain reconstruction	
		• Closed reduction of nasal bone fracture	
Plastic surgery	• Exeresis and suture of small epitheliomas and small benign skin lesions	• Breast reconstruction after total (mastectomy) or partial (quadrantectomy) removal for oncological reasons, with placement of artificial prostheses. Treatment of breast malformations (asymmetries, tuberous breasts, tubular breasts, etc). Treatment of gynecomastia. Breast augmentation. Mastopexy.	• Post-mastectomy breast reconstruction with microsurgical flap. Breast reduction.
	• Scar correction	• Liposuction of abdomen, lower and upper limbs, of intermediate entity.	• Liposuction of abdomen, lower and upper limbs, of considerable entity.
	• Treatment of soft tissue pathologies of the hand	• Functional treatment of trauma outcomes (road accidents, surgical interventions, etc), losses of post-demolition substance of an intermediate entity of face, peri-ocular region (eyelid scars with functional alterations), peribuccal, lower and upper limbs, by means of local flap, skin	• Functional treatment of trauma outcomes (accidents roads, outcomes of surgical interventions, etc), losses of post-demolition substance of considerable entity of face, lower and upper limbs, of the abdomen, of back, by means of flaps, microsurgical or pluritissutal pedunculated transposition flaps of considerable entity

		graft techniques, associated or not with use of artificial dermal substitute	
	• Upper blepharoplasty, lower blepharoplasty	• Lower limb ulcer treatment	• Lower limb ulcer treatment (ASA Class V - IV - III)
	• Otoplasty	• Correction of scars and depressions (lipofilling) of moderate entity.	• Correction of scars and depressions (lipofilling) of considerable entity.
		• Surgical treatment of burns (10% <X <15%).	• Surgical treatment of burns (>15%).
		• Lifting	• Abdominoplasty, post bariatric surgery
			• Removal of large tumors of the soft tissues of the face and neck and plastic reconstruction using a microsurgical flap.
**Maxillofacial surgery**	• Closed reduction of zygomatic arch fracture, closed reduction of mandibular fracture	• Implantology and oral surgery, orthognathic surgery	• Demolition and reconstructive oncological surgery of the head and neck area
	• Lipofilling	• Parotidectomy	• Open reduction of orbitozygomatic fracture
	• Arthrocentesis e temporomandibular joint arthroscopy	• Closed reduction of nasal bone fracture, open reduction of jaw's fracture	• Submandibular sialoadenectomy
	• Cutaneous oncological surgery	• Facial resuscitation in acute and chronic paralysis	
**Neurosurgery**	• Cranial neurosurgery: external ventricular lead, intraventricular catheter for intracranial pressure monitoring	• Spinal neurosurgery: laminectomy> 2spaces, spinal arthrodesis (any type)	• Spinal and cranial neurosurgery: removal of intradural lesions (intracerebral tumors, intraparenchymal haemorrhage)
	• Spinal neurosurgery: herniated disc, laminectomy (≤2 spaces) without arthrodesis	• Cranial neurosurgery: ventriculoperitoneal shunt, extradural lesion removal	
**Vascular intervention procedures**	• Non-tunnelled CVC placement	• CVC tunneled – Port positioning	• TIPS
	• Access for dialysis	• Angiography	
	• CVC removal, Caval filter positioning monitoring	• Caval filter extraction	
	• PICC positioning	• Chemoembolization, uterine embolization (leiomyomas)	
		• Trans-jugular liver biopsy	
**Other intervention procedures**	• Drainage replacement (biliary, nephrostomy, abdominal)	• Non-superficial percutaneous biopsies(excluding kidney and liver)	• Percutaneous kidney and liver biopsy
	• Paracentesis, thoracentesis	• Drainage of deep abscesses (abdominal, hepatic)	• Percutaneous interventional urinary tract
	• Needle aspirations of superficial organs (thyroid, lymph nodes, salivary cells, breast)	• Renal and hepatic thermo ablation	• Percutaneous biliary intervention
	• Aspirations, drainages and surface biopsies	• Cholecystostomy, gastrostomy	
	• Intra-articular aspiration/injection		
**Anaesthesia**	• Superficial blocks: femoral/sciatic/popliteal nerves	• Epidural with catheter, single-shot epidural	• None
	• Fascial blocs: ilioinguinal, iliohypogastric	• Position of deliquorator catheter	
	• Injection into trigger zones	• Spinal anaesthesia	
		• Paravertebral blocks, deep blocks: sympathetic, celiac, stellate ganglion	

CVC, central venous catheters; EGDS, esophagogastroduodenoscopy; ERCP, endoscopic retrograde cholangiopancreatography; EVAR, endovascular aneurysm repair; FESS, functional endoscopic sinus surgery; FNA, fine needle biopsy; PEG, percutaneous endoscopic gastrostomy; PICC, peripherally inserted central catheter
PTA, percutaneous transluminal angioplasty; TIPS, trans-jugular intrahepatic porto-systemic shunt; TURBT, transurethral resection of bladder tumor; TURP, transurethral resection of prostate.
